# Gap Phenomenon of an Abstract Willmore Type Functional of Hypersurface in Unit Sphere

**DOI:** 10.1155/2014/697132

**Published:** 2014-06-05

**Authors:** Yanqi Zhu, Jin Liu, Guohua Wu

**Affiliations:** College of Information System and Management, National University of Defense Technology, Changsha, Hunan 410073, China

## Abstract

For an *n*-dimensional hypersurface in unit sphere, we introduce an abstract Willmore type called *W*_(*n*,*F*)_-Willmore functional, which generalizes the well-known classic Willmore functional. Its critical point is called the *W*_(*n*,*F*)_-Willmore hypersurface, for which the variational equation and Simons' type integral equalities are obtained. Moreover, we construct a few examples of *W*_(*n*,*F*)_-Willmore hypersurface and give a gap phenomenon characterization by use of our integral formula.

## 1. Introduction

Let *φ* : *M* ^*n*^ → *S* ^*n*+1^(1) be an *n*-dimensional compact without boundary hypersurface in unit sphere. Choose an orthonormal frames field {*e*_1_,…, *e*_*n*_, *e*_*n*+1_} along *M* such that {*e*_1_,…, *e*_*n*_} are tangent to *M* and {*e*_*n*+1_} is normal to *M*. Their dual frames are {*θ* ^1^,…, *θ* ^*n*^} and {*θ* ^*n*+1^}, respectively; obviously, *θ* ^*n*+1^ = 0 when it is restricted over *M*. Let *h* denote the second fundamental form of the immersion *φ* : *M* → *S* ^*n*+1^(1). Write

(1) $$\begin{array}{c} h=h_{ij}\theta^{i}⊗\theta^{j} \end{array}$$

and define

(2) $$\begin{array}{c} H=\frac{1}{n}\overset{n}{\underset{i=1}{\sum }}h_{ii},\;\;S=\underset{ij}{\sum }‍{\left(h_{ij}\right)}^{2},\\ \rho =S-nH^{2},\;\;P_{k}=tr\left(h^{k}\right). \end{array}$$

One calls *H*, *S*, *P*_*k*_ to be mean curvature, square of the length, and *k*th power polynomial of second fundamental form, respectively. It is well known that hypersurface *φ* : *M* ^*n*^ → *S* ^*n*+1^(1) is minimal when *H* ≡ 0 and is totally geodesic when *S* ≡ 0. For invariant *ρ*, it is obvious that *ρ*(*Q*) ≥ 0 for all *Q* ∈ *M*, *ρ*(*Q*) = 0 if and only if *Q* is an umbilical point of *M*, and 0 ≤ *ρ*(*Q*) ≤ *C* for all *Q* ∈ *M* and some positive constant *C* since *M* is compact.

In differential geometry, there is a famous classical Willmore functional of hypersurface which is defined as

(3) $$\begin{array}{c} W_{\left(n,n/2\right)}\left(\varphi \right)=\int_{M}\rho^{n/2}dv. \end{array}$$

See [1–4] for the details. It is well known that this functional is invariant under conformal transformation. Its critical point is called classical Willmore or *W*_(*n*,*n*/2)_-Willmore hypersurface. The famous Willmore conjecture says that *W*_(2,1)_(*φ*) ≥ 4*π* ^2^ holds for all immersed tori *φ* : *M* → *S* ^3^; recently, it has been completely solved by Marques and Neves in article [5] using Min-Max theory for two dimensional case, but higher dimensional case is still an open problem. When *n* ≥ 3, the functional *W*_(*n*,*n*/2)_(*φ*) was studied extensively in [6–11].

Due to the importance of Willmore conjecture, many geometric experts generalized the classic Willmore functional to a wide range and some interesting results have been obtained.

In [12], Cai studied the *W*_(2,*p*)_-Willmore functional for surfaces in 3-dimensional unit sphere *S* ^3^(1):

(4) $$\begin{array}{c} W_{\left(2,p\right)}\left(\varphi \right)=\int_{M}{\left(k_{1}-k_{2}\right)}^{p}dv, \end{array}$$

where *k*_1_ and *k*_2_ denote the principal curvatures of *M* and *k*_1_ ≥ *k*_2_. Under some proper conditions, Cai obtained some interesting inequalities.

In [13], Guo and Li considered the *W*_(*n*,1)_-Willmore functional for submanifolds in unit sphere *S* ^*n*+1^(1):

(5) $$\begin{array}{c} W_{\left(n,1\right)}\left(\varphi \right)=\int_{M}\rho dv. \end{array}$$

They calculated the first variation and obtained Simons' type inequalities and finally classified the point-wise gap phenomenon. In [14], Xu and Yang derived the global but not point-wise gap phenomenon for the same functional.

In [15], Wu researched the *W*_(*n*,*r*)_-Willmore functional for submanifolds in unit sphere *S* ^*n*+1^(1):

(6) $$\begin{array}{c} W_{\left(n,r\right)}\left(\varphi \right)=\int_{M}\rho^{r}dv. \end{array}$$

The first variation formula, Simons' type inequalities, and point-wise gap phenomenon are obtained.

Motivated by the work mentioned above, in this paper, we study an abstract Willmore type called *W*_(*n*,*F*)_-Willmore functional:

(7) $$\begin{array}{c} W_{\left(n,F\right)}\left(\varphi \right)=\int_{M}F\left(\rho \right)dv, \end{array}$$

where we always assume that the abstract function satisfies

(8) $$\begin{array}{c} F:u⟶F\left(u\right),\;F\in C^{3}\left(\left[0,+\infty \right)⟶R\right). \end{array}$$

Obviously, functionals *W*_(*n*,*n*/2)_,  *W*_(2,*p*)_,  *W*_(*n*,1)_, and *W*_(*n*,*r*)_ are special cases of *W*_(*n*,*F*)_, so the construction and investigation of *W*_(*n*,*F*)_ are meaningful. As usual, the critical point of functional *W*_(*n*,*F*)_(*φ*) is called *W*_(*n*,*F*)_-Willmore hypersurface. In Section 3, we calculate the 1st variation formula of functional *W*_(*n*,*F*)_.

**Theorem 1**

Let *φ* : *M* ^*n*^ → *S* ^*n*+1^(1) be an *n*-dimensional hypersurface in an (*n* + 1)-dimensional unit sphere *S* ^*n*+1^(1); then *M* is a *W*_(*n*,*F*)_-Willmore hypersurface if and only if

(9) ∑ij(F′(ρ)hij),ji−Δ(F′(ρ)H) +(P3−SH)F′(ρ)−n2F(ρ)H=0.

In particular, if *M* is an isoparametric (all principal curvatures are constant) hypersurface, then *M* is a *W*_(*n*,*F*)_-Willmore hypersurface if and only if

(10) $$\begin{array}{c} \left(P_{3}-SH\right)F^{\prime }\left(\rho \right)-\frac{n}{2}F\left(\rho \right)H=0. \end{array}$$

**Remark 2**

When *F*(*ρ*) = *ρ* ^*n*/2^, *ρ*, *ρ* ^*r*^, Theorem 1 coincides with corresponding results in [6, 13, 15], respectively.

It is well known that Simons' integral inequality plays an important role in the study of minimal hypersurface. It says that if *M* is an *n*-dimensional compact minimal hypersurface in (*n* + 1)-dimensional unit sphere *S* ^*n*+1^(1), then

(11) $$\begin{array}{c} \int_{M}S\left(n-S\right)dv\leq 0. \end{array}$$

For classic Willmore hypersurface, Li [6] proved a Simons' type integral result which says if *M* is an *n*-dimensional compact classical Willmore hypersurface in unit sphere *S* ^*n*+1^(1), then

(12) $$\begin{array}{c} \int_{M}\rho^{n/2}\left(n-\rho \right)dv\leq 0. \end{array}$$

Similarly, for functionals *W*_(*n*,1)_ and *W*_(*n*,*r*)_, [13, 15] obtained integral inequalities, respectively. A natural question is that whether a Simons' type inequality or equality can be established for *W*_(*n*,*F*)_-Willmore functional. We give a confirm answer in this study.

**Theorem 3**

Let *M* be an *n*-dimensional compact *W*_(*n*,*F*)_-Willmore hypersurface in sphere *S* ^*n*+1^(1); then we have Simons' type equality and can give a discussion according to the sign of *F*, *F*′, and *F*′′:

(13) 0=∫MF′′(ρ)|∇ρ|2+2F′(ρ)(|∇h|2−n|∇H|2)  −2ρ(ρ−n)F′(ρ)+nH2(nF(ρ)−2ρF′(ρ))dv.

(1) When *ρ* ≡ *n*, there holds

(14) 0=∫M2F′(n)(|∇h|2−n|∇H|2)   +nH2(nF(n)−2nF′(n))dv.

(2) When *ρ* ≡ 0, there holds

(15) $$\begin{array}{c} 0=\int_{M}2F^{\prime }\left(0\right)\left({\left|\nabla h\right|}^{2}-n{\left|\nabla H\right|}^{2}\right)+n^{2}H^{2}F\left(0\right)dv. \end{array}$$

(3) When *F*′ ≡ 0, *F* ≡ *c* ≠ 0, there holds

(16) $$\begin{array}{c} 0=\int_{M}cn^{2}H^{2}dv. \end{array}$$

(4) When *nF* − 2*uF*′ ≥ 0, *F*′ ≥ 0, *F*′′ ≥ 0, there holds

(17) ∫M2ρ(ρ−n)F′(ρ) =∫MF′′(ρ)|∇ρ|2+2F′(ρ)(|∇h|2−n|∇H|2)   +nH2(nF(ρ)−2ρF′(ρ))dv.

(5) When *nF* − 2*uF*′ ≤ 0, *F*′ ≥ 0, *F*′′ ≥ 0, there holds

(18) ∫M2ρ(ρ−n)F′(ρ)−nH2(nF(ρ)−2ρF′(ρ)) =∫MF′′(ρ)|∇ρ|2+2F′(ρ)(|∇h|2−n|∇H|2)dv.

**Remark 4**

When *F*(*ρ*) = *ρ* ^*n*/2^, *ρ*, *ρ* ^*r*^, Theorem 3 coincides with corresponding results in [6, 13, 15], respectively. Similar equalities also hold for the other sign cases of *F*, *F*′, and *F*′′.

Using the integral equalities in Theorem 3, together with two famous Chern et al. results in [16], we can obtain some conclusions of gap phenomenon.

**Theorem 5**

Let *M* be an *n*-dimensional closed *W*_(*n*,*F*)_-Willmore hypersurface in unit sphere *S* ^*n*+1^(1), we have the following.

1. When *F*′ ≡ 0, *F* ≡ *c* ≠ 0 over (0, +*∞*), if 0 ≤ *ρ* ≤ *n*, then *ρ* = 0 or *ρ* = *n*.
  - For *ρ* = 0, then *H* = 0, *ρ* = 0, *S* = 0, and *M* is totally geodesic;
  - For *ρ* = *n*, then *H* = 0, *ρ* = *n*, *S* = *n*, and $M=C_{m,n-m}=S^{m}(\sqrt{m/n})\times S^{n-m}(\sqrt{\left(n-m\right)/n})$ for some *m* with 1 ≤ *m* ≤ *n* − 1.
2. When *nF* − 2*uF*′ > 0,  *F*′ > 0,  *F*′′ ≥ 0 over (0, *∞*), if 0 ≤ *ρ* ≤ *n*, then *ρ* = 0 or *ρ* = *n*.
  - For *ρ* = 0, then *M* is totally umbilical;
  - For *ρ* = *n*, then *n* ≡ 0(mod⁡  2), *H* = 0, *ρ* = *n*, *S* = *n*, and $M=C_{n/2,n/2}=S^{n/2}(\sqrt{1/2})\times S^{n/2}(\sqrt{1/2})$.
3. When *nF* − 2*uF*′ = 0, *F*′ > 0, *F*′′ ≥ 0 over (0, +*∞*), if 0 ≤ *ρ* ≤ *n*, then *F*(*u*) = *cu* ^*n*/2^, *c* > 0, *ρ* = 0, or *ρ* = *n*.
  - For *ρ* = 0, *M* is totally umbilical;
  - For *ρ* = *n*, $M=W_{m,n-m}=S^{m}(\sqrt{(n-m)/n})\times S^{n-m}(\sqrt{m/n})$ for some *m* with 1 ≤ *m* ≤ *n* − 1.
4. When *nF* − 2*uF*′ < 0, *F*′ > 0, *F*′′ ≥ 0 over (0, +*∞*), if *H* = 0, 0 ≤ *ρ* ≤ *n*, then *H* = 0, *ρ* = 0 or *H* = 0, *ρ* = *n*.
  - For *H* = 0, *ρ* = 0, then *M* is totally geodesic;
  - For *H* = 0, *ρ* = *n*, then *n* ≡ 0(mod⁡  2), *H* = 0, *ρ* = *n*, *S* = *n*, $M=C_{n/2,n/2}=S^{n/2}(\sqrt{1/2})\times S^{n/2}(\sqrt{1/2})$.

**Remark 6**

When *F*(*ρ*) = *ρ* ^*n*/2^, *ρ*, *ρ* ^*r*^, Theorem 5 coincides with corresponding results in [6, 13, 15], respectively. Similar conclusions also hold for the other sign cases of *F*, *F*′, and *F*′′. The examples appeared in Theorem 5 would be constructed in Section 3.

## 2. Preliminaries

Let *φ* : *M* ^*n*^ → *S* ^*n*+1^(1) be an *n*-dimensional closed hypersurface in an (*n* + 1)-dimensional unit sphere and let Φ(·, ·) : *M* × (−*ϵ*, *ϵ*) → *S* ^*n*+1^(1) be a variation of *φ*; it means that

(19) $$\begin{array}{c} \varphi_{t}=:\Phi \left(\cdot ,t\right):M\times \left\{t\right\}⟶S^{n+1}\left(1\right),\;\forall t\in \left(-\epsilon ,\epsilon \right) \end{array}$$

is still an isometric immersion with *φ*_0_ ≡ *φ*.

Let *s* = (*s*_1_,…, *s*_*n*_, *s*_*n*+1_) and *σ* = (*σ* ^1^,…, *σ* ^*n*^, *σ* ^*n*+1^) be the orthonormal local frames of *TS* ^*n*+1^(1) and T*S ^*n*+1^(1), respectively; then *e* = (*e*_1_,…, *e*_*n*_, *e*_*n*+1_) = *φ*_*t*_^−1^ *s* is the orthonormal local frames of the pullback vector bundle *φ*_*t*_^−1^ *TS* ^*n*+1^(1) over *M* × {*t*}, such that {*e*_1_,…, *e*_*n*_} are tangent to *M* and {*e*_*n*+1_} is normal to *M* due to

(20) $$\begin{array}{c} \varphi_{t}^{-1}TS^{n+1}\left(1\right)=TM_{t}⊕T^{⊥}M_{t}. \end{array}$$

Use *ω* to denote the connection form over *TS* ^*n*+1^(1); by the pullback operation, we have the following decomposition:

(21) $$\begin{array}{c} \Phi^{*}\sigma =\theta +Vdt,\;\;\Phi^{*}\sigma^{i}=\theta^{i}+V^{i}dt,\\ \Phi^{*}\sigma^{n+1}=V^{n+1}dt=:fdt,\\ \Phi^{*}\omega =\phi +Ldt,\;\;\Phi^{*}\omega_{i}^{j}=\phi_{i}^{j}+L_{i}^{j}dt,\\ \Phi^{*}\omega_{i}^{n+1}=\phi_{i}^{n+1}+b_{i}dt. \end{array}$$

Thus, one can derive that {*θ* ^*i*^} are the orthonormal frames of T*M and *θ* ^*n*+1^ ≡ 0 when it is restricted over *M*, {*V* ^*i*^, *V* ^*n*+1^ = :*f*} are the variation vector fields of Φ, {*ϕ*_*i*_^*j*^} is the connection form of *TM*, and {*ϕ*_*i*_^*n*+1^ = *h*_*ij*_ *θ* ^*j*^} is the second fundamental form. In particular, we have

(22) $$\begin{array}{c} {\frac{\partial }{\partial t}\varphi_{t}|}_{t=0}=\underset{i}{\sum }V^{i}e_{i}+fe_{n+1}. \end{array}$$

Use *Ω* and *Ω* ^*⊤*^ to denote curvature forms of *TS* ^*n*+1^(1) and *TM*, respectively and write their components:

(23) $$\begin{array}{c} \Omega_{AB}=\frac{1}{2}{\bar{R}}_{ABCD}\sigma^{C}\wedge \sigma^{D},\;A,B,C,D\in \left\{1,\ldots ,n+1\right\},\\ \Omega_{ij}^{⊤}=\frac{1}{2}R_{ijkl}\theta^{k}\wedge \theta^{l},\;i,j,k,l\in \left\{1,2,\ldots ,n\right\}. \end{array}$$

From any one standard differential geometry book, we know that the curvature of unit sphere is

(24) $$\begin{array}{c} {\bar{R}}_{ABCD}=-\left(\delta_{AC}\delta_{BD}-\delta_{AD}\delta_{BC}\right). \end{array}$$

Using connection form {*ϕ*_*i*_^*j*^}, the covariant derivatives of *h*_*ij*_, *H*, *ρ*, *V* ^*i*^, *f*, and *b*_*i*_ can be defined as

(2) $$\begin{array}{c} h_{ij,k}\theta^{k}=d_{M}h_{ij}-\underset{p}{\sum }h_{pj}\phi_{i}^{p}-\underset{p}{\sum }h_{ip}\phi_{j}^{p},\\ h_{ij,kl}\theta^{l}=d_{M}h_{ij,k}-\underset{p}{\sum }h_{pj,k}\phi_{i}^{p}-\underset{p}{\sum }h_{ip,k}\phi_{j}^{p}-\underset{p}{\sum }h_{ij,p}\phi_{k}^{p},\\ H_{,i}\theta^{i}=d_{M}H,\;\;H_{,ij}\theta^{j}=d_{M}H_{,i}-\underset{p}{\sum }H_{,p}\phi_{i}^{p},\\ \Delta H=\underset{i}{\sum }H_{,ii},\\ \rho_{,i}\theta^{i}=d_{M}\rho ,\;\;\rho_{,ij}\theta^{j}=d_{M}\rho_{,i}-\underset{p}{\sum }\rho_{,p}\phi_{i}^{p},\;\;\Delta \rho =\underset{i}{\sum }\rho_{,ii},\\ V_{,j}^{i}\theta^{j}=d_{M}V^{i}+\underset{p}{\sum }V^{p}\phi_{p}^{i},\\ V_{,jk}^{i}\theta^{k}=d_{M}V_{,j}^{i}-\underset{p}{\sum }V_{,p}^{i}\phi_{j}^{p}+\underset{p}{\sum }V_{,j}^{p}\phi_{p}^{i},\\ f_{,i}\theta^{i}=d_{M}f,\;\;f_{,ij}\theta^{j}=d_{M}f_{,i}-\underset{p}{\sum }f_{,p}\phi_{i}^{p},\;\;\Delta f=\sum f_{,ii},\\ b_{i,j}\theta^{j}=d_{M}b_{i}-\underset{p}{\sum }b_{p}\phi_{i}^{p},\\ b_{i,jk}\theta^{k}=d_{M}b_{i,j}-\underset{p}{\sum }b_{p,j}\phi_{i}^{p}-\underset{p}{\sum }b_{i,p}\phi_{j}^{p}. \end{array}$$

We use *d*_*M*_,  *d*_*M*×(−*ϵ*,*ϵ*)_ = *d*_*M*_ + *dt*(∂/∂*t*) and *d* to denote the differential operators on *M*, *M* × (−*ϵ*, *ϵ*), and *S* ^*n*+1^(1), respectively. By calculating directly and comparing both sides of the following equations:

(26) $$\begin{array}{c} \Omega =d\omega -\omega \wedge \omega ,\;\;0=d\sigma -\sigma \wedge \omega , \end{array}$$

(27) $$\begin{array}{c} \Phi^{*}\left(\Omega \right)=d_{M\times (-\epsilon ,\epsilon )}\Phi^{*}\left(\omega \right)-\Phi^{*}\left(\omega \right)\wedge \Phi^{*}\left(\omega \right), \end{array}$$

(28) $$\begin{array}{c} 0=d_{M\times (-\epsilon ,\epsilon )}\Phi^{*}\left(\sigma \right)-\Phi^{*}\left(\sigma \right)\wedge \Phi^{*}\left(\omega \right), \end{array}$$

we would derive out the lemmas below; also see article [10]. In fact, the right hand side of (27) is

(29) Φ∗(ΩAB)=Φ∗(12R¯ABCDσC∧σD)=12R¯ABCDΦ∗(σC)∧Φ∗(σD)=12R¯ABCDθC∧θD+dt∧(R¯ABCDVCθD),

while the left hand side of (27) is

(30) dM×(−ϵ,ϵ)Φ∗(ωAB)−Φ∗(ωAC)∧Φ∗(ωCB) =(dM+dt∂∂t)(ϕAB+dtLAB)−(ϕAC+dtLAC)(ϕCB+dtLCB) =(dMϕAB−ϕAC∧ϕCB)  +dt(∂∂tϕAB−(dMLAB+LACϕCB−ϕACLCB));

comparing (29) with (30), we obtain

(31) $$\begin{array}{c} \left(d_{M}\phi_{A}^{B}-\phi_{A}^{C}\wedge \phi_{C}^{B}\right)=\frac{1}{2}{\bar{R}}_{ABCD}\theta^{C}\wedge \theta^{D},\\ \frac{\partial }{\partial t}\phi_{A}^{B}-\left(d_{M}L_{A}^{B}+L_{A}^{C}\phi_{C}^{B}-\phi_{A}^{C}L_{C}^{B}\right)={\bar{R}}_{ABCD}V^{C}\theta^{D}. \end{array}$$

The equation of (28) is

(32) 0=dM×(−ϵ,ϵ)Φ∗(σ)−Φ∗(σ)∧Φ∗(ω)=(dM+dt∂∂t)(θ+dtV)−(θ+dtV)∧(ϕ+dtL)=dMθ−θ∧ϕ+dt(∂θ∂t−dMV−Vϕ+θL).

Comparing both sides, we obtain

(33) $$\begin{array}{c} d_{M}\theta -\theta \wedge \phi =0,\;\;\frac{\partial \theta }{\partial t}-d_{M}V-V\phi +\theta L=0. \end{array}$$

From (31) and (33), we can easily conclude the following lemmas which are useful in the variation calculation and establishment of Simons' type integral equalities.

**Lemma 7**

Let *φ* : *M* ^*n*^ → *S* ^*n*+1^(1) be a hypersurface; one has structure equations:

(34) $$\begin{array}{c} R_{ijkl}=-\left(\delta_{ik}\delta_{jl}-\delta_{il}\delta_{jk}\right)-\left(h_{ik}h_{jl}-h_{il}h_{jk}\right),\;h_{ij,k}=h_{ik,j}. \end{array}$$

**Lemma 8**

Let *φ* : *M* ^*n*^ → *S* ^*n*+1^(1) be a hypersurface; one has Ricci identity:

(35) hij,kl−hij,lk=−(δikhjl−δilhjk)−(δjkhil−δjlhik) −(hikhplhpj−hilhpkhpj) −(hjkhplhip−hjlhpkhip).  

**Lemma 9**

Let *φ* : *M* ^*n*^ → *S* ^*n*+1^(1) be a hypersurface and let *V* = *V* ^*⊤*^ + *V* ^⊥^ = *V* ^*i*^ *e*_*i*_ + *fe*_*n*+1_ be a variation vector field; one has

(36) ∂θi∂t=(V,ji−Lji−hijf)θj,∂hij∂t=f,ij+∑phij,pVp+∑phpjLip+∑phipLjp+∑phiphpjf+δijf.

**Lemma 10**

With the same notations as above, one has

(37) $$\begin{array}{c} \underset{ijk}{\sum }h_{ik}h_{kj}L_{j}^{i}=0,\;\;\underset{ij}{\sum }h_{ij}L_{j}^{i}=0. \end{array}$$

## 3. Variation Calculation and Examples

To calculate the first variation of *W*_(*n*,*F*)_-Willmore functional, we need two lemmas.

**Lemma 11**

Let *φ* : *M* ^*n*^ → *S* ^*n*+1^(1) be a hypersurface and let *V* = *V* ^*⊤*^ + *V* ^⊥^ = *V* ^*i*^ *e*_*i*_ + *fe*_*n*+1_ be a variation vector field. Suppose that *dv* = *θ* ^1^∧⋯∧*θ* ^*n*^ denotes the volume element; one has

(38) $$\begin{array}{c} \frac{\partial dv}{\partial t}=\underset{i}{\sum }V_{,i}^{i}-nHf. \end{array}$$

**Proof**

By Lemma 9, we have (∂/∂*t*)(*θ* ^*i*^) = (*V*_,*j*_^*i*^ − *L*_*j*_^*i*^ − *h*_*ij*_ *f*)*θ* ^*j*^. Hence,

(39) $$\begin{array}{c} \frac{\partial dv}{\partial t}=\underset{i}{\sum }\theta^{1}\wedge \cdots \frac{\partial }{\partial t}\left(\theta^{i}\right)\cdots \wedge \theta^{n}=\underset{i}{\sum }V_{,i}^{i}-nHf. \end{array}$$

With this, we complete the proof of Lemma 11.

**Lemma 12**

Let *φ* : *M* ^*n*^ → *S* ^*n*+1^(1) be a hypersurface and let *V* = *V* ^*⊤*^ + *V* ^⊥^ = *V* ^*i*^ *e*_*i*_ + *fe*_*n*+1_ be a variation vector field; one has

(40) $$\begin{array}{c} \frac{\partial \rho }{\partial t}=\underset{ij}{\sum }2h_{ij}f_{,ij}-2H\Delta f+2P_{3}f-2SHf+\underset{i}{\sum }\rho_{,i}V^{i}. \end{array}$$

**Proof**

By the definition of *ρ*,  *S* and *H*, one has

(41) $$\begin{array}{c} \frac{\partial }{\partial t}\left(\rho \right)=\frac{\partial }{\partial t}\left(S\right)-\frac{\partial }{\partial t}\left(nH^{2}\right). \end{array}$$

It follows Lemmas 9 and 10 that

(42) ∂∂t(S)=2∑ijhij∂∂t(hij)=2∑ijhij[f,ij+∑phij,pVp+∑phpjLip      +∑phiPLjp+∑phiphpjf+δijf]=2∑ijhijf,ij+∑iS,iVi+2P3f+2nHf.

By Lemmas 9 and 10 again, one has

(43) ∂∂t(nH2)=2nH∂∂tH=2H∑i[f,ii+∑phii,pVp+∑phpiLip      +∑phpiLip+∑phiphpif+δiif]=2HΔf+∑i(nH2),iVi+2HSf+2nHf.

Hence,

(44) $$\begin{array}{c} \frac{\partial \rho }{\partial t}=\underset{ij}{\sum }2h_{ij}f_{,ij}-2H\Delta f+2P_{3}f-2HSf+\underset{i}{\sum }\rho_{,i}V^{i}. \end{array}$$

With this, we complete the proof of Lemma 12.

**Proof of Theorem 1**

By Lemmas 11 and 12, we can calculate

(45) ∂∂tW(n,F)(φt)=∫MtF′(ρ)∂∂t(ρ)+F(ρ)(∑iV,ii−nHf)dv=∫MtF′(ρ)(∑ij2hijf,ij−2HΔf+2P3f−2SHf+∑iρ,iVi)  +F(ρ)(∑iV,ii−nHf)dv=∫Mt2[∑ij(F′(ρ)hij),ji−Δ(F′(ρ)H)     +(P3−SH)F′(ρ)−n2HF(ρ)]f dv.

With this, we complete the proof of Theorem 1.

From Theorem 1, we can find a few examples in unit sphere *S* ^*n*+1^(1), in particular, the isoparametric hypersurfaces. Since all principal curvatures *k*_1_,…, *k*_*n*_ are constant, then *ρ*, *H*, *S* are constant and *H* = (1/*n*)*P*_1_, *S* = *P*_2_, *ρ* = *P*_2_ − (1/*n*)(*P*_1_)^2^. Thus, *W*_(*n*,*F*)_-Willmore hypersurface equation becomes

(46) $$\begin{array}{c} \left(P_{3}-\frac{1}{n}P_{1}P_{2}\right)F^{\prime }\left(\rho \right)-\frac{1}{2}P_{1}F\left(\rho \right)=0. \end{array}$$

**Example 13**

Obviously, totally geodesic hypersurfaces are *W*_(*n*,*F*)_-Willmore for any abstract function *F*, totally umbilical but not totally geodesic hypersurfaces are *W*_(*n*,*F*)_-Willmore if and only if *F*(0) = 0.

**Example 14**

For a particular hypersurface with *n* ≡ 0(mod⁡2),

(47) $$\begin{array}{c} C_{n/2,n/2}=S^{n/2}\left(\frac{1}{\sqrt{2}}\right)\times S^{n/2}\left(\frac{1}{\sqrt{2}}\right)⟶S^{n+1}\left(1\right). \end{array}$$

All principal curvatures are

(48) $$\begin{array}{c} k_{1}=\cdots =k_{n/2}=1,\;\;k_{(n/2)+1}=\cdots =k_{n}=-1. \end{array}$$

We can derive the quantities *P*_1_,  *P*_2_,  *P*_3_,  *ρ*, respectively, *P*_1_ = 0,  *P*_2_ = *n*,  *P*_3_ = 0,  *ρ* = *n*. Obviously *C*_*n*/2,*n*/2_ always is a *W*_(*n*,*F*)_-Willmore hypersurface of *S* ^*n*+1^(1) for any function *F*.

**Example 15**

For a family hypersurfaces with parameters *λ*,  *μ*,  0 < *λ*, *μ* < 1, *λ* ^2^ + *μ* ^2^ = 1.

(49) $$\begin{array}{c} S^{m}\left(\lambda \right)\times S^{n-m}\left(\mu \right)⟶S^{n+1}\left(1\right),\;1\leq m\leq n-1. \end{array}$$

Obviously, all principal curvatures are

(50) $$\begin{array}{c} k_{1}=\cdots =k_{m}=\frac{\mu }{\lambda },\;\;k_{m+1}=\cdots =k_{n}=-\frac{\lambda }{\mu }. \end{array}$$

Then the quantities *P*_1_, *P*_2_, *P*_3_, *ρ* are, respectively,

(51) $$\begin{array}{c} P_{1}=m\frac{\mu }{\lambda }-\left(n-m\right)\frac{\lambda }{\mu },\;\;P_{2}=m\frac{\mu^{2}}{\lambda^{2}}+\left(n-m\right)\frac{\lambda^{2}}{\mu^{2}},\\ P_{3}=m\frac{\mu^{3}}{\lambda^{3}}-\left(n-m\right)\frac{\lambda^{3}}{\mu^{3}},\\ \rho =\frac{m\left(n-m\right)}{n}\left[\frac{\mu^{2}}{\lambda^{2}}+\frac{\lambda^{2}}{\mu^{2}}+2\right]. \end{array}$$

Setting *μ*/*λ* = *x* > 0, *W*_(*n*,*F*)_-Willmore hypersurface equation becomes

(52) 2m(n−m)F′(ρ)x6−m[nF(ρ)−2(n−m)F′(ρ)]x4 +(n−m)[nF(ρ)−2mF′(ρ)]x2 −2m(n−m)F′(ρ)=0.

**Example 16 (Clifford torus [16]; *F*(*ρ*) ≡ 1)**

Consider that

(53) $$\begin{array}{c} C_{m,n-m}=S^{m}\left(\sqrt{\frac{m}{n}}\right)\times S^{n-m}\left(\sqrt{\frac{n-m}{n}}\right),\;1\leq m\leq n-1 \end{array}$$

are minimal hypersurfaces with *H* ≡ 0,  *S* ≡ *n*,  *ρ* ≡ *n*.

**Example 17 (classic Willmore torus [6]; *F*(*ρ*) = *ρ* ^*n*/2^)**

Consider that

(54) $$\begin{array}{c} W_{m,n-m}=S^{m}\left(\sqrt{\frac{n-m}{n}}\right)\times S^{n-m}\left(\sqrt{\frac{m}{n}}\right),\;1\leq m\leq n-1 \end{array}$$

are *W*_(*n*,*n*/2)_-Willmore hypersurfaces with *ρ* = *n*. When some *W*_*m*,*n*−*m*_ is minimal, then *n* ≡ 0(mod⁡  2), *m* = *n*/2.

## 4. Simons' Type Equalities and Gap Phenomenon

Some lemmas are needed for the establishment of Simons' type integral equalities and the discussion of gap phenomenon.

**Lemma 18**

For *ρ*, one has

(55) Δρ=∑ij2nhijH,ij−2nHΔH+2|∇h|2 −2n|∇H|2+2nρ+2nHP3−2S2,

where |∇*h*|^2^ = ∑_*ijk*_ *h*_*ij*,*k*_^2^.

**Proof**

By the definition of *ρ* and Laplacian operator, we have

(56) Δρ=∑k(∑ijhij2−nH2),kk=∑k(∑ij2hijhij,k−2nHH,k),k=∑k(∑ij2hij,k2+∑ij2hijhij,kk−2nH,k2−2nHH,kk)=2|∇h|2−2nHΔH−2n|∇H|2+2∑ijkhijhij,kk=2(|∇h|2−nHΔH−n|∇H|2+(T1)∑ijkhijhij,kk),

for term *T*_1_ = ∑_*ijk*_ *h*_*ij*_ *h*_*ij*,*kk*_; by Lemmas 7 and 8, easily we obtain

(57) T1=∑ijkhijhij,kk=∑ijkhijhik,jk=∑ijkhij(hik,jk−hik,kj+hik,kj)=∑ijkhijhkk,ij+∑ijkhij(hik,jk−hik,kj)=∑ijnhijH,ij+(T2)∑ijkhij(hik,jk−hik,kj),

for term *T*_2_ = ∑_*ijk*_ *h*_*ij*_(*h*_*ik*,*jk*_ − *h*_*ik*,*kj*_); by Lemma 8, we have

(58) T2=∑ijkhij(hik,jk−hik,kj)=∑ijkhij{[−(δjkhik−δkkhij)−(hjkhiphpk−hkkhiphpj)]      +[−(δijhkk−δikhjk)−(hijhkphpk−hikhkphpj)]}=−S+nS−∑ijkphijhjkhiphpk+nHP3−n2H2 +S−S2+∑ijkphijhikhkphpj=nρ−S2+nHP3.

Substituting (58) into (57), we have

(59) $$\begin{array}{c} T_{1}=\underset{ij}{\sum }nh_{ij}H_{,ij}+n\rho -S^{2}+nHP_{3}. \end{array}$$

Substituting (59) into (56), we have

(60) Δρ=∑ij2nhijH,ij−2nHΔH+2|∇h|2 −2n|∇H|2+2nρ+2nHP3−2S2.

With this, we complete the proof of Lemma 18.

**Lemma 19 (Huisken's estimate [17])**

Consider that

(61) $$\begin{array}{c} {\left|\nabla h\right|}^{2}\geq \frac{3n^{2}}{n+2}{\left|\nabla H\right|}^{2}\geq n{\left|\nabla H\right|}^{2}, \end{array}$$

and |∇*h*|^2^ = *n* | ∇*H*|^2^ if and only if ∇*h* = 0.

**Proof**

We decompose the tensor ∇*h* as follows:

(62) $$\begin{array}{c} h_{ij,k}=E_{ijk}+F_{ijk}, \end{array}$$

where

(63) $$\begin{array}{c} E_{ijk}=\frac{n}{n+2}\left(H_{,i}\delta_{jk}+H_{,j}\delta_{ik}+H_{,k}\delta_{ij}\right),\\ F_{ijk}=h_{ij,k}-E_{ijk}. \end{array}$$

Through a computation, we obtain

(64) $$\begin{array}{c} {\left|E\right|}^{2}=\frac{3n^{2}}{n+2}{\left|\nabla H\right|}^{2},\;\langle E,F\rangle =0. \end{array}$$

Finally, by the triangle inequality, we have

(65) $$\begin{array}{c} {\left|\nabla h\right|}^{2}\geq {\left|E\right|}^{2}=\frac{3n^{2}}{n+2}{\left|\nabla H\right|}^{2}\geq n{\left|\nabla H\right|}^{2}. \end{array}$$

If |∇*h*|^2^ = *n* | ∇*H*|^2^, then all inequalities become equalities, *F*_*ijk*_ = 0, ∇*H* = 0, and *E*_*ijk*_ = 0; thus, ∇*h* = 0. With this, we complete the proof of Lemma 19.

**Lemma 20**

For *F*(*ρ*), one has

(66) ΔF(ρ)=F′′(ρ)|∇ρ|2+2F′(ρ)(|∇h|2−n|∇H|2) −2ρ(ρ−n)F′(ρ)+nH2(nF(ρ)−2ρF′(ρ)) +2n[∑ijF′(ρ)hijH,ij−F′(ρ)HΔH      +(P3−SH)F′(ρ)H−n2F(ρ)H2].

**Proof**

We know that

(67) $$\begin{array}{c} \Delta F\left(\rho \right)=F^{\prime \prime }\left(\rho \right){\left|\nabla \rho \right|}^{2}+F^{\prime }\left(\rho \right)\Delta \rho . \end{array}$$

By Lemmas 18 and 19, we can easily obtain the identity.

**Proof of Theorem 3**

Integrating the equality in Lemma 20 over *M*, we have

(68) 0=∫MF′′(ρ)|∇ρ|2+2F′(ρ)(|∇h|2−n|∇H|2)  −2ρ(ρ−n)F′(ρ)+nH2(nF(ρ)−2ρF′(ρ))  +2n[∑ijF′(ρ)hijH,ij−F′(ρ)HΔH      +(P3−SH)F′(ρ)H−n2F(ρ)H2].

By Theorem 1, we know that

(69) ∑ij(F′(ρ)hij),ji−Δ(F′(ρ)H) +(P3−SH)F′(ρ)−n2F(ρ)H=0.

Using Stokes theorem, we obtain

(70) 0=∫MF′′(ρ)|∇ρ|2+2F′(ρ)(|∇h|2−n|∇H|2)  −2ρ(ρ−n)F′(ρ)+nH2(nF(ρ)−2ρF′(ρ)).

We complete the proof of Theorem 3.

In order to prove Theorem 5, we need two very important conclusions which are treated as a lemma and a main Theorem in Chern et al.'s article [16]. For a hypersurface, we choose frame fields in such a way that *h*_*ij*_ = 0, for all  *i* ≠ *j*,  *h*_*i*_ = *h*_*ii*_.

**Lemma 21 (Lemma 3 of [16])**

Let *x* : *M* ^*n*^ → *S* ^*n*+1^(1) be a compact hypersurface with ∇*h* ≡ 0; then there are two cases.

1. *h*_1_ = ⋯ = *h*_*n*_ = *λ* = *constant*, and *M* is a totally umbilic (*λ* > 0) or totally geodesic (*λ* = 0);
2. *h*_1_ = ⋯*h*_*m*_ = *λ* = *constant* > 0, *h*_*m*+1_ = ⋯ = *h*_*n*_ = −(1/*λ*), 1 ≤ *m* ≤ *n* − 1, and *M* is a Riemannian product of *M*_1_ × *M*_2_, where $M_{1}=S^{m}(1/\sqrt{1+\lambda^{2}})$,$\;\;M_{2}=S^{n-m}(\lambda /\sqrt{1+\lambda^{2}})$.

**Lemma 22 (main Theorem of [16])**

Clifford torus *C*_*m*,*n*−*m*_, 1 ≤ *m* ≤ *n* − 1 are the only compact minimal (*H* = 0) hypersurfaces of dimension *n* in unit sphere *S* ^*n*+1^(1) satisfying *S* = *n*.

**Proof of Theorem 5**

Obviously, case (1) of Theorem 5 is a corollary of Lemma 22; thus, we just focus cases (2) and (3); the rest can be proved by similar argument.

(2) When *nF* − 2*uF*′ > 0,  *F*′ > 0,  *F*′′ ≥ 0 over (0, +*∞*), by the case (4) of Theorem 3, we have

(71) ∫M2ρ(ρ−n)F′(ρ) =∫MF′′(ρ)|∇ρ|2+2F′(ρ)(|∇h|2−n|∇H|2)   +nH2(nF(ρ)−2ρF′(ρ))≥0.

If 0 ≤ *ρ* ≤ *n*, the right hand side of identity (71) is nonpositive, while the left hand side of (71) is nonnegative; thus, we can conclude that *ρ* = 0 or *ρ* = *n*. For *ρ* = 0, *M* is totally umbilical; for *ρ* = *n*, substituting it into the above equality together with Lemma 19, we can derive ∇*h* = 0, *H* = 0, *ρ* = *n*, and *S* = *n*, by Lemma 22, we know that *M* = *C*_*m*,*n*−*m*_; moreover, by *W*_(*n*,*F*)_-Willmore equation, we conclude that *n* ≡ 0(mod⁡  2), *H* = 0, *ρ* = *n*, *S* = *n*, and *M* = *C*_*n*/2,*n*/2_.

(3) When *nF* − 2*uF*′ = 0, *F*′ > 0, *F*′′ ≥ 0  (0, +*∞*), we know that *F*(*u*) = *cu* ^*n*/2^, *c* = const > 0. By the case (4) of Theorem 3, we have

(72) ∫Mρn/2(ρ−n) =∫Mn−24|∇ρ|2+ρ(n/2)−1(|∇h|2−n|∇H|2)≥0.

Moreover, if 0 ≤ *ρ* ≤ *n*, then *ρ* = 0 or *ρ* = *n*. For *ρ* = 0, *M* is totally umbilical; for *ρ* = *n*, substituting it into the above equality together with Lemma 19, we obtain ∇*h* = 0; by Lemma 22, we know that *M* is a torus; then, by Example 17, we can conclude that *M* is a classic Willmore torus *W*_*m*,*n*−*m*_ for some *m*.
